# Perceived challenges and enablers to evaluating a whole systems approach initiative: Reflections of embedded researchers

**DOI:** 10.1371/journal.pone.0324174

**Published:** 2025-05-27

**Authors:** Lauren Clifford, Adam Mars, Oliver Hamer

**Affiliations:** 1 Sport, Physical Activity, Health and Wellbeing Research Group, Department of Sport and Physical Activity, Edge Hill University, Ormskirk, Lancashire, United Kingdom; 2 Evaluation and Policy Analysis Unit, Faculty of Health, Social Care and Medicine, Edge Hill University, Ormskirk, Lancashire, United Kingdom; Indian Institute of Technology Madras, INDIA

## Abstract

Physical inactivity remains a substantial public health concern, with complex socio-environmental factors contributing to increasing inactivity. Whole systems approaches to physical activity seek to address these complexities by promoting multi-component, place-based interventions. This study reflects on the experiences of three embedded researchers working within a whole systems approach initiative aimed at reducing physical inactivity in the United Kingdom. Researchers were embedded within a local authority and affiliated to a university whilst responsible for evaluating the effectiveness and efficacy of the whole systems approach initiative. Using a reflective journaling method, followed by inductive thematic analysis, the findings identified key challenges and enablers to evaluating the initiative. Key challenges included the perceived value of research and evaluation within the local authority, a lack of capacity to conduct evaluative activity, and the presence of confirmation and reporting bias within the wider delivery team. Key enablers included relationship-building, skill development, and protected time for evaluation and research activity. The findings suggest that institutions supporting embedded researchers should establish regular contact with the local authority, help to establish realistic expectations, and support researchers to overcome emerging challenges. Recommendations for researchers include developing robust relationships, setting out clear expectations, and ensure they have protected time at key points during the evaluation. Through these recommendations, researchers may be better prepared to overcome implementation challenges and improve the efficiency of the evaluation process.

## Introduction

Physical inactivity remains a critical public health challenge [1], with the global age-standardised prevalence of inactivity estimated to be 31% [2]. Despite concerted efforts by several key organisations and government, epidemiological data suggests that inactivity levels are rising, particularly in western countries [3]. Recent findings suggest that rates of inactivity within the UK are high, with the highest of these rates in the North of England (approximately 25% of the population) [4]. Given rising rates, it could be suggested that traditional intervention approaches, characterised by a singular focus on individual-level behaviour change (short-term, < 12 weeks), have not been wholly effective in achieving sustained behaviour change (>24-months) [5]. This may be because physical activity and inactivity are influenced by the complex interplay of individual, biological, sociopolitical, societal and environmental factors [6,7]. Acknowledging these complexities, whole systems approaches (WSAs) have emerged as a promising alternative to traditional short term interventions [8]. WSAs consider the multifaceted nature of physical inactivity and seek to implement coordinated, multi-sectoral strategies that address policy, environmental, and organisational factors, alongside individual behaviours [9,10]. Modifications to policy and environments which directly and indirectly relate to physical activity (within wider systems), is likely to have more success in changing how people live, work, and interact [11,12]. By moving beyond isolated interventions and considering the broader systemic influences on activity levels, WSAs offer a pathway to more sustainable and long-term change [13,14].

A growing body of evidence advocates for the adoption of multi-component WSAs to promote physical activity at the population level [15–17]. This approach places a greater emphasis on understanding and responding to the needs of the local population and because of this, are sometimes referred to as place-based (joined-up approach that considers the ‘place’, and not just individual concerns) [18,19]. WSAs consider how determinants of the ‘place’ (i.e., individual motivations, capacity, self-efficacy, infrastructure, policy, and environment) may interact to influence physical activity behaviour [20–22]. By embedding opportunities for physical activity within broader social and environmental structures, WSAs ensure that movement becomes an integrated aspect of everyday life [21,22]. To achieve integration, literature suggests that WSAs require strong leadership who are willing to adopt new ways of working, collaborative partnerships to enable resource-sharing and alignment across sectors, and community engagement to encourage community ownership (improving sustainability) [23].

Although WSAs designed to reduce physical inactivity have had some success, evaluation of complex systems-wide interventions has been found to be challenging [24–26]. This is because they require a nuanced understanding of the local context and the existing systems of the place (including its processes and practises) [19,26]. In addition, several barriers exist that often prevent evaluation from establishing the effectiveness of the approach [26–29]. Such barriers include; difficulties measuring intended outcomes (e.g., population physical activity levels), multiple components operating at different systems levels (e.g., delivery of physical activity in the community and influencing leadership within networks), the absence of control groups for comparative purposes, the extensive resources needed to measure outcomes over the longer term (i.e., 5–10 year follow up), and the multitude of economic and social changes that occur in a place which often impacts behaviour change (e.g., political and industrial shifts) [24,26–28].

One approach which has been used to mitigate many of these barriers to WSA evaluations is the use of embedded researchers [24,29,30]. Embedded researchers have been defined as those who work within a host organisation who are not often research active, whilst also maintaining affiliation with an academic institution [30,31]. The key benefit to this approach is that researchers can access contextual information within an organisation (and the place), gain an understanding of the ways of working (i.e., processes), identify how they collaborate with system partners, co-produce research which fits with the organisations aims, and build research capacity for the organisation (closely aligned to their services) [29,32]. However, there are several challenges faced by embedded researchers, particularly in complex whole system interventions. Within clinical settings, barriers such as a lack of funding, poor research infrastructure, inadequate support from leadership and issues with dual association have been found to have an impact on the success of the approach [29]. With that said, there is little known about the barriers faced by embedded researchers in other sectors such as local authority, education, or leisure [29]. Consequently, there is a need to understand the barriers other researchers face when embedded within these sectors to inform the implementation of future projects and embedded researcher schemes [33].

## Materials and methods

Using a reflective journaling method (outlined by Lutz and Paretti), followed by inductive Thematic Analysis (detailed by Braun and Clarke), the aim of this paper was to present reflections from three embedded researchers evaluating a WSA initiative named ‘Together an Active Future’ (TaAF) [34–36].

### Embedded researchers

The author team includes three embedded researchers (two male, one female). Two researchers were employed by a higher education academic institution (Edge Hill University) working in partnership with Blackburn with Darwen Borough Council. One researcher was employed by Blackburn with Darwen Borough Council and affiliated to Edge Hill University. All three researchers had extensive experience in research and evaluation, two holding doctorate degrees (PhD’s) whilst one had a Master’s degree (MSc). All three researchers had full time roles evaluating the WSA initiative which aimed to tackle physical inactivity and reduce physical activity inequalities. All three researchers had been embedded in the WSA initiative for at least six months prior to the data collection. Each researcher was working on different intervention arms (termed workstreams) within the WSA initiative, but met weekly to share insights. Table 1 provides further detailed information about the embedded researcher roles, and relevant interventions/workstreams.

**Table 1:** Information about the WSA, the embedded researcher roles, and workstreams.

About the WSA	
**Name:** Together an Active Future. **Initiated:** December 2017. **Footprint:** East Lancashire, England; including the six districts of Blackburn with Darwen, Burnley, Hyndburn, Pendle, Ribble Valley and Rossendale. Approximate target population of 575,852 [37]. **Vision:** TaAF is a collective of people who come together from different organisations and backgrounds to change the way things work. TaAF aims to establish locally driven cross-policy considerations for tackling physical activity inequalities. **Principles:** TaAF takes a whole-systems approach to tackling physical inactivity. Collaboration with local people and partners is fundamental to the work. Central to TaAF’s model for change is the idea that people who are changing themselves (attitude, behaviours, priorities) are the key to our systems and places changing for the better. The model for change is underpinned by systems science and behaviour change theory (COM-B and Theory of Planned Behaviour) [38,39]. **Key partners:** Local authorities, active partnerships, educational institutions, NHS Trusts, voluntary and community organisations, leisure services, elected officials and local businesses. **Host organisation:** Blackburn with Darwen Borough Council.	
**Researcher: LC**	
**About the** **workstream**	**About the** **embedded researcher and role**
**Name of workstream:** Active Schools (Ready Set Move). **Aim:** To work with school leaders and middle leaders to embed physical activity and movement within the school day through a whole-school approach. **Programme:** Middle Leaders Programme A leadership development programme for teachers who have aspirations of senior leadership. It provides investment into their personal and professional development and empowers them to lead change across their own school. Across a series of training and development workshops, schools work towards making changes to their own practice and whole school policies. Using the Creating Active Schools Framework [40], schools will review, assess, and evaluate their existing physical activity provision. This allows them to plan for and make sustainable changes to school practice and policy, making physical activity and movement easier for all young people to take part in. The programme aims to: Create more physically active schools across Pennine Lancashire. Increase physical activity levels in children and young people. Develop leadership skills of middle leaders. Create a network of middle leaders who will champion physical activity across the school network.	**Evaluation period:** May 2023 – May 2025 **Embedded role:** LC is embedded in TaAF’s Active Schools workstream on a full-time basis based at Edge Hill University. **Nature of the role:** To develop and evaluate the utility of the active schools’ approaches for the promotion of whole-school physical activity for children & young people across East Lancashire. Being embedded within the TaAF team meant that LC was able to understand the intricacies of TaAF’S whole-systems place-based approach and the Active Schools workstream. As a result of being embedded, LC was able to: Build and maintain relationships with TaAF colleagues and school staff. Facilitate programme delivery and embed evaluation into the workshops. Co-create a research protocol with Middle Leaders to capture child-level data. Attend meetings, gather insight, observe proceedings, and implement quantitative and qualitative data collection, and carry out analysis. Provide meaningful feedback to schools through a continuous learning cycle. Disseminate research findings to various audiences.
**Researcher: AM**	
**About the** **workstream**	**About the** **embedded researcher and role**
**Name of workstream:** Active Madrassahs. **Aim:** To work with Islamic leaders and teachers working in madrassahs (supplementary schooling with faith based Islamic curriculum) to create more opportunities to undertake physical activity for children and young people who attend madrassah. **Programmes:** The Active Madrassahs workstream is a multi-component workstream that looks to create improvements in physical activity participation for Muslim children and young people. The Active Madrassah workstream aims to achieve the following: Take a place-based approach to physical activity promotion through brokering relationships with Islamic leaders to co- produce approaches to physical activity promotion in their settings. With the support of national governing bodies, to upskill and enhance resources for madrassahs to improve their physical activity offer. Embed opportunities for play and physical activity in madrassah time by improving knowledge of physically active pedagogy. Increase levels of physical activity for Muslim children and young people.	**Evaluation period:** March 2024 - March 2025 **Embedded role:** AM is employed by Edge Hill University whilst also working in an embedded way with TaAF on the Madrassah workstream. **Nature of the role:** To evaluate and explore the barriers and enablers of embedding physically active learning and physical activity promotion in madrassahs across East Lancashire. To use the principles of co–production to ensure that cultural and religious sensitivity are embedded throughout the research. The findings will help practitioners understand vital learning on what has worked well in the workstream, impacting subsequent rounds of practice and resource allocation. AM was able to: Maintain regular communication with workstream lead. Conduct rigorous qualitative data collection with a arrange of stakeholders. Analyse data from interviews, sense checking and interpreting findings in collaboration with stakeholders in the workstream. Communicate findings in both formal and informal ways using storytelling, visuals, and other traditional presentations of data. Produce detailed reports and published outputs on the findings of the workstream to various audiences.
**Researcher: OH**	
**About the** **workstream**	**About the** **embedded researcher and role**
**Name of workstream:** Active networks. **Aim:** To increase levels of physical activity among 6 districts of East Lancashire. **Programmes:** Active BwD Network, Rossendale Connected, Hyndburn Way, Pendle Active Place, Community Action Network, Active Burnley Forum. There have been several networks set up to facilitate collaborations across multiple system partners who promote physical activity across East Lancashire. These networks bring together partners who have a shared vision of increasing physical activity of people within their place. The programme aims to: Get more people more active across East Lancashire. Improve the health and wellbeing of people in East Lancashire through reducing inactivity. Increase physical activity opportunities for people in East Lancashire. Create a large network of stakeholders who will champion physical activity within their place.	**Evaluation period:** November 2023 to March 2025. **Embedded role:** OH is embedded in TaAF’s Local Network workstreams on a full-time basis based within Blackburn with Darwen Borough Council, affiliated to Edge Hill University. **Nature of the role:** To evaluate local network workstreams to understand the impact of the TaAF whole-systems place-based approach within the six districts of East Lancashire. OH was able to: Collaborate with intervention leads to develop a comprehensive evaluation protocol designed to capture detailed data. Attend relevant meetings to gather insights, observe proceedings, and implement rigorous quantitative and qualitative data collection methods. Conduct thorough analysis of collected data to generate actionable insights. Assist in designing and implementing research methodologies that aligned with the WSA to activity. Write detailed reports and presentations to disseminate findings and recommendations. Ensure all research activities complied with ethical standards and data protection regulations.

### The whole systems approach initiative

Together an Active Future is a multi-component, complex, physical activity initiative developed with the aim of tackling physical inactivity and physical activity inequalities across East Lancashire. The initiative was developed as a WSA, engaging systems partners in its implementation and delivery. The initiative was funded by Sport England’s Local Delivery Pilot Programme. To date, TaAF has conducted place-based activities involving partnership building, physical activity sessions, creative engagement, capacity building, education, and training [35]. These activities have prompted a differentiated approach creating opportunities for, and participation in physical activity [35]. Since its inception, TaAF has delivered a total of 43 interventions (known within the initiative as workstreams). These workstreams have focused on integrating physical activity across sectors/ systems, strengthening community capacity for activity, and tackling structural inequalities [35]. To deliver these workstreams, TaAF has established working partnerships with influential leaders made up of chief executive officers from local NHS Trusts, public health leads within local authorities, managers of local leisure trusts, elected members of local government, head teachers within schools, professors of HE institutions and members of community and voluntary sector services. Examples of these workstreams include Creative Football, a workstream focused on engaging men with mental health concerns in non-competitive football activities; Burnley Outdoor Town, a community wide campaign which engages community members and local leadership to make better use of Burnley’s green and blue spaces for activity; Active Practice, a health system intervention which places health coaches within GP practises to provide support and guidance to patients and staff; and a series of active schools/ madrassah leadership programs that support the integration of physical activity into the learning environment for children and young people [35]. Table 1 provides further detailed information about the WSA.

### Data collection

We employed a reflective journaling method outlined by Lutz and Paretti, to systematically collect data relating to our embedded researcher reflections [36]. Initially, each embedded researcher independently documented their reflections of the WSA weekly within a reflective journal over a period of twelve weeks (during the summer of 2024) [36]. Whilst being guided by the focus of being an embedded researcher, this period of reflection was largely unstructured to enable the individual researcher to reflect on their own experiences without being constrained by a specific research question. However, we often reflected on important events, key challenges, new developments within the initiative, the evaluation process, and the key factors that enabled us to be effective within our roles.

Microsoft Word (version 2408) was used independently by each researcher to document their reflections. The embedded researchers did not discuss their reflections with each other until the first data analysis session which occurred 2 weeks after the data collection period had concluded. The TaAF initiative was approximately four years into a five-year funded programme when data collection for this study began.

### Data analysis

The data analysis followed Braun and Clarke’s approach to reflexive inductive thematic analysis [34]. In step one, the three researchers convened face-to-face to discuss individual reflections, familiarising themselves with what had been documented during the data collection process. In step two, all three researchers independently coded the data to identify commonalities and differences, which were then systematically charted (using Microsoft Excel, version 2408). Following the coding, the researchers independently documented some suggestions for initial themes. In step three, the researchers convened face-to-face for a second time to individually present their coding and outline their thoughts on some initial themes. Following this, there was a group discussion reviewing the initial themes that had emerged from the data collection. In step four, all three researchers independently reviewed the themes and defined a label for each. In step five, the researchers met for a third and final time to refine and name the themes. Any disagreements were discussed at length until agreement was reached. Following discussion, the researchers reached a consensus on the final set of themes and a thematic map was generated. In step six, the researchers collectively wrote up the findings. All researchers reviewed the findings and approved the final version of the manuscript.

As part of the data analysis, each of the researchers reflected on some key parts of reflexivity. They discussed their length of time in role, stage of career, and experiences within the role that may have possibly shaped the reflections.

## Results

**Fig 1:**
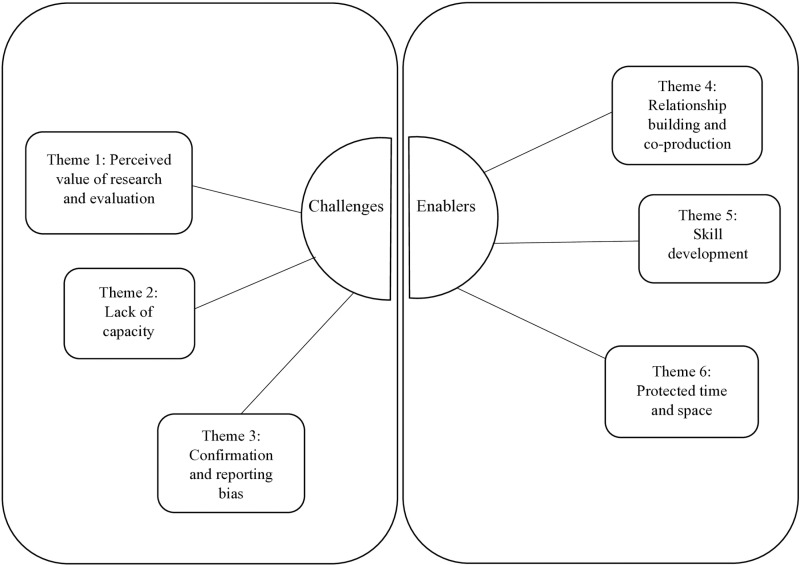
Thematic map. Thematic map visually displaying the themes of researcher’s reflections.

In total, six themes emerged that highlighted the challenges and enablers to evaluating a WSA initiative to reducing inactivity. We identified three key themes that we perceived to be a challenge to being embedded and conducting an evaluation within a WSA: (1) perceived value of research and evaluation; (2) lack of capacity; (3) confirmation and reporting bias. In addition, we also identified three key themes that we perceived to be enablers to being embedded and conducting an evaluation within a WSA: (4) relationship building and co-production; (5) skill development; and (6) protecting time and space. A thematic map was developed which can be seen in Fig 1.

### Theme 1. Challenge - Perceived value of research and evaluation

We reflected on the challenges of being embedded researchers evaluating within a host organisation who are facilitating a WSA to reducing physical inactivity. From the commencement of our roles, it was clear that evaluation was not embedded from the early stages.

*“One of the key things that I’ve noticed about evaluation within a local authority and embedded within community physical activity interventions, is that evaluation is often an afterthought. There are no evaluation strategies that are implemented or considered within a developed design phase of interventions. This often prevents any baseline data from being collected and often all evaluation is done retrospectively. This gave me the perception that evaluation wasn’t particularly valued.”* (Embedded researcher 3).

The perceived lack of value for research and evaluation made it difficult for researchers to become embedded within the environment, and to start collecting data with individuals (e.g., programme staff and stakeholders) who had no prior experience of evaluation.

In addition to the perceived lack of value for evaluation, we reflected on the challenges associated with the adaptive, responsive nature of WSA that we had to navigate as embedded researchers. For example, all evaluation findings had to be available in a timely manner to maximise their use by key stakeholders and workstream leads. This process of formative evaluation allowed colleagues involved in the delivery of the work to adapt their practice to improve the effectiveness of their work. However, this urgency to report findings back at regular intervals led to a regular practise of reporting only preliminary results (lacking a comprehensive analysis).

*“People within the team seem very comfortable with receiving ideas that are unpolished, not always well thought out and iterative. This is a benefit of moving at pace where a greater level of error is tolerated in communication, thoughts, and expression so that continual progress is made. On some occasions however this has led to superficial reporting of evidence.”* (Embedded researcher 2).

High quality research and evaluation requires extensive time, and this is not always understood or valued by individuals working outside of higher education (e.g., local authority or third sector). As highlighted above, the host organisation was comfortable with receiving preliminary results so that continuous progress can be made to their delivery. However, this approach to evaluation is contrary to typical research practice. For instance, the preliminary results could in fact change once time has been given to rigorous data analysis. This process of reporting only preliminary findings could damage relationships with key stakeholders, and result in colleagues within the host organisation devaluing evaluation due to inconsistent messaging (disseminated from the findings).

In addition to a disparity in perceptions about the value of evaluation, a further reflection was that local authority staff had very limited knowledge of research methods and methodology.

*“There is a real challenge with basic research knowledge among intervention leads… Outside of some loose qualitative methods there was a lack of research methodology or rigorous methods within the local delivery pilot. There are very little quantitative measures and the qualitative methods are largely reliant upon team reflections rather than stakeholder engagement.”* (Embedded researcher 3).

As previously highlighted, there was a lack of evaluation culture within the host organisation, and the processes for data collection (in place) were not systematic or conducted using rigorous methods. Data was typically collected through reflective practice and qualitative methods with no quantitative outcome data to support the narratives. This made it challenging to establish overall effectiveness of the initiative.

In summary, the reflections in this theme highlight the challenges associated with a perceived lack of value for research and evaluation that embedded researchers face within local authorities.

### Theme 2. Challenge - Lack of capacity

Whole system approaches to physical activity, have significant financial and resource investment which allows the work to grow rapidly. Much of the resource investment is allocated to the main delivery of the work; however, evaluation capacity does not increase to match the growth of the work.

*“When reflecting on why evaluation does not seem to be a key part of local authority working, it is obvious that resources are allocated to the delivery of interventions without consideration for evaluation. Conducting rigorous evaluation takes resource and capacity and although there is some recognition of this, it does not take priority over delivery. Essentially conducting rigorous evaluation of the interventions would require resources moving away from the delivery toward the evaluation, in which the organisation do not deem necessary.”* (Embedded researcher 3).

We reflected on the lack of evaluation capacity and how the growth of the work has come with an expansion of responsibility and expectation which was not associated with the roles in the early days of employment (e.g., contributing to events organisation and workshops). This often led to feelings of being overwhelmed and being pulled away from the core responsibilities of our roles.

In addition, because of the nature of being embedded within the delivery of the initiative whilst also holding a position within a higher education institute, there were challenges with capacity that meant it was difficult to meet with line managers or supervisors. This often exacerbated feelings of being overwhelmed.

*“I have felt very ‘in between’ two teams and organisations. … This is particularly challenging when whole-system approaches are fast-paced as the academic members of staff who are involved in the project are not always available to meet due to their academic responsibilities.”* (Embedded researcher 2).

We considered the adaptive, dynamic and fast-paced nature of WSAs and the challenge this brings for researchers with regards to data analysis. As discussed above, there was a lack of capacity and resource for evaluation because the delivery of the work took priority. Due to this and the responsive nature of WSAs, a lot of data are collected at various time points, but there is little capacity to rigorously analyse it:

*“The lack of capacity and resource for evaluation within a whole-systems approach runs the risk of collecting a lot of data, which runs a further risk of that data sitting dormant and not being analysed.”* (Embedded researcher 1).

Reflecting on the theme, researchers discussed the importance of analysing and disseminating all the data that was collected to share learning and insights with other researchers working in a similar space.

In summary, the reflections in this theme highlight that while significant resources are invested in program delivery, evaluation efforts often lack sufficient resources. This can lead to a disconnect between delivery and evaluation priorities.

### Theme 3: Challenge – Confirmation and reporting bias

We reflected on our place as embedded researchers within an organisation that is often exclusively focused on identifying data which portrays the success of an initiative. This focus is unsurprising given the need to secure future funding to continue the initiative. Whilst the need to continually secure funding is a reality for many organisations working in this sector, it sometimes felt like findings which showed that some parts of the initiative may have been ineffective, were not given adequate consideration:

*“Working as an embedded researcher comes with its difficulties because the organisation in which you are hosted in, has both a financial and resource investment in the intervention that you are working to evaluate. There was a tendency to overlook data that does not support the success of the intervention.”* (Embedded researcher 3).

We reflected on how the host organisation was sometimes challenged about the initiative’s effectiveness and how the data could be employed to identify ineffective components. We also reflected on how data which showed no improvements in outcomes could be mobilised to prevent wasting of resources. However, the complex nature of the initiative and lack of resource for evaluation made it challenging to confirm resources were directed toward effective components/ solutions.

At the cultural level of the organisation, whilst the positive spin on the impact of interventions seems to help drive motivation within the team, we reflected on how this often-diverted attention away from a deeper engagement with the evaluation. A deeper engagement may have highlighted areas of learning to improve the delivery of the workstreams. To maintain the integrity of the research, we were transparent about the findings of the evaluation:

*“It is challenging if the results are interpreted as being unfavourable, as the host organisation typically focus on outputs that demonstrate positive outcomes. This is particularly difficult as a researcher who tries to remain objective and reports on what the data are telling us. Upon reflection, it is important to be transparent if something didn’t work, and to not dismiss this data as it is still a finding, even if it is not a positive one.”* (Embedded researcher 1).

Moreover, we reflected on the way in which elements of this bias (reporting) has shaped the reporting and dissemination of research. There was a tendency to produce outputs which only showcased the positive findings:

*“The outputs the organisation produces are visually appealing and very well organised but there is not a great deal of data connected to them. The style of the outputs often feels journalistic and anecdotal, this certainly makes them more user friendly and engaging however the effect/impact of interventions are often discussed in an optimistic way.*” (Embedded researcher 2).

We reflected on how some outputs may not have truly captured the key themes of the data we had collected. However, we recognised that this reporting bias was not likely intentional but may have been a result of pre-conceived ideas and beliefs (confirmation bias).

In summary, this theme has described the presence of confirmation and reporting bias in the organisation, describing how this influences the culture of the organisation but also the process of decision making made around workstreams and the creation of evaluation related outputs.

### Theme 4: Enabler - Relationship building and co-production

As embedded researchers, we reflected on the key enablers to evaluating a WSA initiative. A key enabler described by all researchers was the process of building strong relationships with the deliverers and intervention leads. These relationships enabled access to data and data collection opportunities that were not available early in the researcher’s role. This was key given that there was a lack of pre-existing data collection processes which needed to be put in place to evaluate outcomes:

*“In addition to a lack of data, locality leads we’re not accustomed to researchers requesting data from them and so it made my job extremely difficult going in to try and collect data. I had to build a very strong relationship with the leads and delivers of the work in order to work successfully with them to collect and analyse data. This required about 3 months’ worth of meeting with all local networks comprehensively understanding what they were doing.”* (Embedded researcher 3).

In addition, forging strong relationships with the deliverers (programme staff) supported the recruitment of key stakeholders to establish the effectiveness of the intervention. This was important because traditional academic and clinical means of recruitment which focus on monetary incentivisation was unsuccessful:

*“There is an important element of being an embedded researcher within a local authority and across leisure trusts in that a strong relationship is required to be able to conduct data collection. Unlike traditional research in healthcare, financial incentive is not enough to convince people to engage because local authorities have substantial budget and other priorities. From my experience, offering financial recompense for participants to engage with research is unsuccessful.”* (Embedded researcher 2).

Not only did strong relationships enable data collection, but it also helped during stages of planning and dissemination:

*“Although building these relationships requires a lot of time, it has been invaluable for the planning, delivery, and dissemination of research outputs.”* (Embedded researcher 1).

Reflecting on this, researchers deemed their relationships, and collaborations as important to ensuring their research was conducted in a timely and robust manner:

*“Upon reflection, had I not spent the time building and maintaining these relationships, the project would not have had so much success in such a short space of time. For instance, stakeholders may not have been so responsive to working with us in an evaluation capacity.”* (Embedded researcher 1).

Further reflections highlighted how a co-production approach to evaluation within a local authority setting differed somewhat from traditional academic research projects. Trust between the researcher and the local authority staff was essential for co-production because there was an initial scepticism from the intervention leads about the motives of the researchers. It was clear that the evaluation would only progress at the pace of perceived trust:

*“Compared to traditional research projects that are commonly associated with inflexible project timelines due to the pressures of grant funding, such time to build these relationships with key stakeholders is not always built into research timelines which can have negative effects on the success of the project, particularly in co-creation research. ‘Co-creation only moves at the speed of trust.’*” (Embedded researcher 1).

In summary, the reflections within this theme highlight that relationship building, and co-production are key enablers that enhance implementation, data collection and dissemination of findings for embedded researchers. By investing some time and effort into forging strong relationships, researchers may be able to overcome logistic challenges, navigate complex processes, enhance recruitment of participants, access existing data and deliver evaluation activities more efficiently.

### Theme 5: Enabler - Skill development

This theme highlights the required skills, learning, and development of embedded researchers who have conducted research and evaluation in a complex, whole systems, physical activity intervention. Reflections focused on the skills which enabled efficient evaluation within a local authority which may differ somewhat from traditional academic and clinical settings.

An important enabler was the necessity to be flexible and adaptable in data collection practices. This is largely because stakeholders are difficult to contact and have little capacity to take part:

*“As an embedded researcher the opportunities for data collection often are not structured or planned. There is a need to be extremely flexible and prepared to conduct data collection at very short notice. This requires a real confidence and experience as a researcher to converse with individuals off the cuff.”* (Embedded researcher 3).

Reflecting on this, a high level of confidence may be required prior to being embedded as there is little time to plan or organise in preparation for an interview, focus group or distribution of a survey.

In addition to being adaptable and flexible, we also reflected on the importance of having a comprehensive understanding of research methodologies to support unplanned data collection. A thorough understanding of research methodologies that are well-suited to capturing the complexity of a WSA (e.g., realist and ethnography) are key:

*“As an embedded researcher, a real focus of data collection is understanding the wider context and development of the interventions within the place. This lends itself to realist and ethnographic methodology which captures stories of behaviour change that relate to the communities. Without a strong methodological understanding, the unplanned data collected may not have provided the rich data needed.”* (Embedded researcher 3).

This reflection highlights the importance of researchers preparing to be embedded, undertaking methodological skill development, particularly in those methodologies that relate to their role.

A further reflection is that we considered the complexity of evaluating a WSA initiative without the resource to conduct large scale quantitative data collection. This meant that we had to adapt and utilise innovative methods to capture the impact of the work. These demands necessitated a rapid cycle of learning and applying new methods which was outside of traditional qualitative and quantitative methods (e.g., interviews, focus groups and surveys):

*“Evaluating within a whole-systems space is demanding, and it challenges us as researchers to be adaptive and responsive to new ways of working. Although it is important that we draw on robust and rigorous quantitative and qualitative methods, whole-system approaches require us to learn about new emerging methods such as Ripple Effects Mapping.”* (Embedded researcher 1).

For embedded researchers working in complex whole systems initiatives, there is an ongoing need to identify and adopt new methods that support evaluation, particularly if there are a lack of resources.

Reflecting on our roles, we also identified communication skills as a key enabler for embedded researchers. All researchers echoed similar thoughts which suggested a greater demand for social interactions than would usually be expected of a researcher in a traditional academic setting. It was valuable to attend networking events, lunchtime clubs and stakeholder workshops to meet new stakeholders. Because of this, there was a need to develop comprehensive social skills for the role:

*“Social skills are invaluable in whole-systems approaches to build trust and rapport and maintain relationships with key stakeholders that can benefit the research process... Other social skills that I feel have been central to my role are empathy, active listening, emotional intelligence, verbal and non-verbal communication, particularly in the language that I use when communicating to different stakeholders.”* (Embedded researcher 1).

In summary, the experience of being an embedded researcher in a WSA to physical activity can often be challenging and require additional skills compared to that of a traditional academic researcher. From these reflections, there has been a need to develop and refine methodological expertise, communication skills and explore innovative data collection and evaluation methods.

### Theme 6: Enabler - Protected time and space

This theme highlights that an embedded researcher’s time and space can be occupied very quickly with activities not related to research and evaluation. The reflections of embedded researchers suggest that routinely protecting time for dedicated periods for reflection, data analysis, and academic output is key to navigating the demands of a local authority setting.

A key reflection was that all researchers perceived the working environment as ‘busy’, and this often was accompanied by many unscheduled interactions:

*“It is sometimes challenging as an embedded researcher because the organisation focuses on communication, networking, relationship building, and partnership working. This environment lends itself to a busy and often manic office space… The social nature of the organisation often detracts from researcher’s time to execute their role as an evaluator.”* (Embedded researcher 3).

A key reflection was how all researchers needed to actively manage the dual demands of university and local authority settings. These demands were often contrasting and each needed to be managed in different ways.

*“There is often the feeling of being pulled away from the core deliverables and outputs that are important to the research which were discussed as part of the role at outset. There are also specific challenges for early career researchers, or those that are coming off the back of the PhD experience, because their PhD programme is likely to have moved at a much slower pace than the work required for the local authority. Ultimately, I think it is important to try and establish boundaries and protect your time as best as possible around core deliverables, realising perhaps that reaching the end of the to-do list and meeting the demands of both university and local authority might be a persistent challenge that you must go with rather than trying to solve.” (Embedded researcher 2).*

This highlights the ongoing tension between a collaborative, fast-paced working environment of a complex WSA intervention, with systematic and rigorous research activity (e.g., data collection and analysis).

There was a collective feeling that although there is a need to adapt to the ongoing needs of the work, it is also important to maintain the integrity of research processes and methodology. Without these, embedded researchers are in danger of becoming journalists, with fewer standards and considerations for the integrity of the data. We reflected on how to manage these challenges, agreeing that protecting time and space was critical for embedded researchers. Regular periods of protected time can assist in maintaining rigorous research and evaluation activity:

*“As a researcher, there is often a need for solitary time to analyse both qualitative and quantitative data. Embedded within an environment which is busy, full of inquisitive questions, and where research expertise is needed more than capacity allows, it is extremely challenging to find solitary time… Protected capacity it is essential to maintain the quality of the research. There is a need for balance where embedded researchers can be responsive but also operate with integrity and rigour.”* (Embedded researcher 3).

In summary, while the dynamic and collaborative nature of complex interventions presents unique challenges for embedded researchers, actively protecting time and space is a key enabler for evaluation. By allocating dedicated time researchers can manage the needs of their roles, ensuring that they can deliver rigorous, methodologically sound research that meets the needs of both the local authority and the higher education institution.

## Discussion

The aim of this paper is to present reflections based on the challenges and enablers of three embedded researchers working within a WSA initiative. Through a systematic approach to the collection and analysis of our reflections, we established six themes that we perceived to be challenges and enablers to being embedded within a WSA. The challenges included: (1) perceived value of research and evaluation; (2) lack of capacity; and (3) confirmation and reporting bias. The enablers included: (4) relationship building and co-production; (5) skill development; and (6) protecting time and space.

The three key challenges largely align with those reported in existing literature [19,29,41]. In particular, there is a strong consensus that researchers embedded in organisations which sit outside of academia (i.e., local authority and clinical settings), will often experience a culture which does not value research and evaluation [29,30]. Researchers can prepare themselves for these cultural differences by taking the time to develop knowledge of the values within the host organisation [41]. Researchers should be prepared to directly and indirectly teach the values of research and evaluation to individuals working within the host organisation, in order to positively influence the culture [30]. In addition to cultural challenges, our findings also align with studies that highlight a lack of capacity as an important barrier for embedded researchers [19,41]. Researchers may need to develop capacity building strategies within host organisations, to avoid becoming involved in non-research related activities (i.e., delivery of interventions, administration etc) [41]. It may also be a benefit for researchers to have an awareness of how capacity issues may cause tensions between practitioners and researchers, given that evaluation is often an additional activity that is not factored into day-to-day workloads [30,41]. Given that challenges related to capacity have been reported to decrease the quality and rigour of research activity, researchers may want to seek additional funding (through targeted funding streams such as the NIHR) in an attempt mitigate these challenges (prior to any evaluation) [41,42].

A further challenge highlighted in our findings, was the concern of confirmation and reporting bias. To our knowledge, this is a novel reflection which has not yet been published in other papers outlining the challenges of undertaking an embedded researcher role. Researchers planning to be embedded within non-academic settings must be prepared to encounter strong beliefs about the effectiveness of an intervention, without there being any robust evidence to support these perceptions. It is likely that researchers will be placed in challenging situations whereby they feel pressured to align their findings with the organisation’s expectations, but it is essential they maintain an objective position to avoid compromising the integrity of the evaluation [43]. Researchers may need to be prepared to challenge confirmation bias, particularly as it portrays to exploring a broad set of outcomes and not being persuaded to only measure those which may lead to positive results [44]. If embedded researchers do not attempt to mitigate confirmation bias, the effectiveness of interventions could be inaccurately portrayed, reducing opportunities for learning and improvement [43]. In addition, a focus on specific outcomes may have a negative impact on decision-making which could see ineffective interventions receive further investment [43].

In addition to the challenges, our findings highlighted several key enablers which can support embedded researchers when evaluating interventions within a local authority setting. These enablers are consistent with the findings of other studies and can provide guidance to prepare researchers to be embedded in the public sector [29]. The findings of this study add to the consensus of literature that emphasises the need for researchers to build robust, positive working relationships within the host organisation (i.e., senior leadership teams and delivery officers of local authorities) [19,29,32,41]. Literature suggests that face-to-face meetings, networking events and a co-design approach to evaluation may help to facilitate trust and strengthen relationships between researchers and practitioners [19,41]. In addition to relationship building, another key enabler was the development of additional skills to assist the researcher during the embedding process. Further skills relating to methodology and communication may prepare them for the unique activities (i.e., upskilling practitioners, mentoring, changing culture etc) that may arise during their role. These findings are consistent with other literature highlighting a need for embedded researchers to be able to upskill public sector workers, communicate research to a range of audiences, enhance organisational culture, effectively manage their own time, and mentor stakeholders to use the data in a way that helps them improve service delivery [19,30,41,45].

### Recommendations

#### Recommendations for host organisation and academic institutions.

***Organise regular meetings to debrief and reflect***: Organise frequent meetings with key stakeholders involved in the evaluation including local authority staff, intervention leads, and researchers. This ensures that all stakeholders are involved in the process of decision making at regular intervals throughout the evaluation. Having frequent, open, and honest communication will allow researchers to keep key stakeholders up to date with progress and buffer any tensions as and when they arise.

***Set expectations and boundaries for the embedded researcher role***: In the early stages key stakeholders involved in the evaluation including commissioners, intervention leads, and the wider academic team should set out their expectations for the embedded researcher role. This can help to establish boundaries around the core deliverables of the role, whilst managing expectations for the evaluation in relation to time frame, resource, and capacity. Acknowledging that it is impossible to capture and evaluate every activity will help deal with the responsive and overwhelming nature of WSAs.

***Be adaptable and flexible***: All parties involved should expect the evaluation approach to adapt given the dynamic, fast-paced nature of WSAs. Understanding this prior to the role can better prepare embedded researchers to respond to collecting data at short notice, whilst being prepared to learn and apply new, non-traditional methods. For the host organisation, there should be an acknowledgement that research may not move at the same pace as practice, and that rigorous evaluation takes time.

### Recommendations for researchers

#### Prioritise building relationships early in the process.

Initially, invest some time to establish and foster relationships with key stakeholders, including local authority staff, intervention leads, and those delivering the interventions. Actively build trust as an enabler to gaining access to participants data and navigating the complexities of the organisation. Take any opportunity to engage with networking events within the local authority to gain a comprehensive understanding of the people and the work.

#### Upholding impartiality.

Embedded researchers should continually reflect on the context they are working in, perhaps keeping a reflexive journal or taking some observational notes on the organisation’s orientation towards research. This will help to identify biases and blind spots in the organisations research culture. Researchers should take any opportunity to showcase the value of the research to decision makers within the host organisation.

#### Protect time and space.

Proactively allocate and protect regular blocks of time which are dedicated to core research activities such as data collection, analysis, and output writing. In addition, communicate the complexity of the research process to stakeholders, highlighting the difference between rapid summaries and rigorous research to manage expectations. Consider the importance of relationship building and networking but manage time to ensure that these activities do not take away from the core evaluation.

#### Being connected to wider teams.

Given the potential lack of extensive research expertise in local authorities, is it important that embedded researchers are connected to and supported by a wider team throughout the evaluation (particularly early career researchers). Being able to draw on the expertise of others can enhance the evaluation and helps to bridge the gap between academia and practice.

### Strengths and limitations

Having reflected on our experiences of being embedded researchers within a WSA, it is important to consider the strengths and limitations of the reflections that we have shared. A strength of this paper is that we have provided unique insights into the role of an embedded researcher within a WSA (local authority host), whilst using a rigorous approach to data collection and analysis (detailed in the methods). Although these reflections are specific to the initiative and workstreams in which we operated, we believe that they will resonate with other embedded researchers working within a WSA, and in turn, provide useful insights for researchers who are considering or embarking on an embedded researcher role. However, we recognise that our paper has some limitations. Firstly, the study only included reflections from three embedded researchers working within the same WSA and therefore, are not representative of wider WSAs. This being said, embedded researcher roles are rare, particularly in local authorities and so these reflections add a novel contribution to literature. We also acknowledge that the reflecting journaling method is inherently subjective and so the interpretation of the findings and recommendations may be influenced by the researcher’s positionality, and their ability to accurately recall events. Furthermore, the embedded researcher reflections were collected during a specific period of the evaluation (years following implementation), and so the insights may not fully capture the dynamic nature of the initiative from its inception.

A further limitation is that the paper does not include reflections of wider stakeholders or other members operating within the host organisation. In doing so, we cannot understand the experiences of those who are commissioning embedded researchers to evaluate WSAs to tackling physical inactivity.

## Conclusion

In conclusion, the findings highlight the key reflections of embedded researchers working as part of a local authority whole systems initiative to reducing physical inactivity. The reflections highlight several key challenges which included: a perceived lack of value for research and evaluation, inadequate capacity, and the presence of confirmation and reporting bias. Conversely, relationship-building, skill development, and protecting time and space were identified as key enablers that could enhance an embedded researcher’s ability to carry out their role. The skill to develop trust with local authority staff, communicate effectively with local stakeholders, conduct unstructured data collection, and maintain a balance between academic rigor and practical responsiveness, were key to being effective within the role. The findings highlight the importance of protected time and space to ensure quality within a non-academic environment which may place pressure on researchers to produce rapid evaluation summaries and discard rigorous methodological processes. The findings suggest that higher education institutes supporting embedded researchers should establish regular contact with the local authority to manage expectations, establish realistic timelines and address emerging challenges of the evaluation process. Embedded researchers may be better equipped to conduct their role if they build robust relationships with the host organisation, whilst also setting out clear expectations for what will be delivered during the evaluation. In addition, embedded researchers may need to prepare themselves to work flexibly and dynamically during key phases of the evaluation, whilst also protecting time for systematic research processes (i.e., data analysis).
